# Strategies for Efficient Computation of the Expected Value of Partial Perfect Information

**DOI:** 10.1177/0272989X13514774

**Published:** 2014-01-21

**Authors:** Jason Madan, Anthony E. Ades, Malcolm Price, Kathryn Maitland, Julie Jemutai, Paul Revill, Nicky J. Welton

**Affiliations:** School of Social and Community Medicine, University of Bristol, Bristol, UK (JM, AEA, MP, NJW); Department of Medicine, Imperial College, London, UK (KM); KEMRI-Wellcome Trust Research Programme, Kilifi, Kenya (KM, JJ); Centre for Health Economics, University of York, York, UK (PR); Warwick Medical School, University of Warwick, Coventry, UK (JM); School of Health and Population Sciences, University of Birmingham, Birmingham, UK (MP)

**Keywords:** value-of-information, Bayesian methods, cost-effectiveness analysis

## Abstract

Expected value of information methods evaluate the potential health benefits that can be obtained from conducting new research to reduce uncertainty in the parameters of a cost-effectiveness analysis model, hence reducing decision uncertainty. Expected value of partial perfect information (EVPPI) provides an upper limit to the health gains that can be obtained from conducting a new study on a subset of parameters in the cost-effectiveness analysis and can therefore be used as a sensitivity analysis to identify parameters that most contribute to decision uncertainty and to help guide decisions around which types of study are of most value to prioritize for funding. A common general approach is to use nested Monte Carlo simulation to obtain an estimate of EVPPI. This approach is computationally intensive, can lead to significant sampling bias if an inadequate number of inner samples are obtained, and incorrect results can be obtained if correlations between parameters are not dealt with appropriately. In this article, we set out a range of methods for estimating EVPPI that avoid the need for nested simulation: reparameterization of the net benefit function, Taylor series approximations, and restricted cubic spline estimation of conditional expectations. For each method, we set out the generalized functional form that net benefit must take for the method to be valid. By specifying this functional form, our methods are able to focus on components of the model in which approximation is required, avoiding the complexities involved in developing statistical approximations for the model as a whole. Our methods also allow for any correlations that might exist between model parameters. We illustrate the methods using an example of fluid resuscitation in African children with severe malaria.

Value of information (VoI) methods provide a coherent decision-theoretic approach to research prioritization.^1,2^ When making a decision under imperfect information, it is possible that the expected optimal decision is wrong, and this possibility will be associated with an expected loss. VoI methods define the value of research in terms of the expected reduction in this expected loss resulting from the additional information gained. This is derived from the expected impact of the proposed study on parameter uncertainty. It is therefore natural to take a Bayesian perspective where prior beliefs about parameters are “updated” by incorporating new evidence to form posterior beliefs.^3^ The expected loss given current information is also known as the expected value of perfect information (EVPI), because it is the amount the decision maker should be willing to pay to eliminate all parameter uncertainty in the decision.^1,2^ Where there are multiple sources of uncertainty, it is possible to calculate the EVPI on a subset of focal parameters, termed the *expected value of partial perfect information*, or alternatively, *expected value of perfect parameter information* (EVPPI). EVPI and EVPPI provide an upper bound to the value of any proposed future study. Several authors have highlighted the use of these methods in health technology assessment in relation to sensitivity analysis and research prioritization, and we refer interested readers to this literature for a more complete discussion of the role of value-of-information analysis in health technology assessment.^4,5^

EVPPI involves an inner expectation nested within an outer expectation, which can be challenging to estimate; a common approach is nested Monte Carlo simulation.^6^ There are 3 main issues with the nested simulation approach. First, obtaining estimates of adequate precision can be computationally expensive.^7^ Second, estimates will have significant upward bias if insufficient simulation sample sizes are used for the inner loop.^8^ Third, it can be difficult to generate unbiased EVPPI estimates when correlations exist between parameters, unless the joint distribution of the correlated parameters takes a known parametric form.^7^ Conditions for the net benefit function have been identified that, if satisfied, allow EVPPI to be calculated in a single step by replacing the nonfocal parameters with their expected means.^9^ In this article, we extend the work of Ades and others,^9^ describing methods that can be used to avoid nested simulation in a broad range of situations, through careful consideration of the structure of the net benefit function and the relationships between its parameters. We illustrate these methods using a case study involving fluid resuscitation in African children with severe malaria, followed by a discussion of the advantages of the methods compared with other approaches and limitations resulting from the assumptions made.

## Motivating Example

Malaria accounts for up to 1 million deaths annually in children younger than 5 years living in Sub-Saharan Africa. Children hospitalized with cerebral malaria (coma) have increased case fatality (18%–21%),^10^ and survivors are at heightened risk of long-term neurological sequelae (NS).^11,12^ There are clinical arguments suggesting that fluid resuscitation could reduce mortality in African children.^13^ Furthermore, pilot studies, involving 3 fluids (saline, albumin, and gelofusine), provided limited but promising support for this hypothesis.^13–15^ Following these studies, a large multinational trial, the FEAST trial, was initiated to provide definitive evidence on the efficacy of fluid resuscitation.^16^

We developed an economic model to assess the cost-effectiveness of fluid resuscitation strategies, taking a health services perspective in which the relevant costs and benefits are those relating to the individual patient (irrespective of who actually provides the funds for health care). The model is based on the assumptions given in Table 1, which were informed by consultation with clinical experts advising the FEAST trial. Assumption A6, used to derive the probability of NS in those receiving fluids, requires further explanation. If fluid resuscitation reduces mortality, there will be a subgroup of survivors who would have died without fluids. Assumption A6 allows for the possibility that this subgroup is more severely affected by malaria than those who would have survived without fluids and therefore are at greater risk of NS. Note that we do not assume this a priori; the model allows for the possibility that fluids increase mortality or that improved survival is associated with reduced NS risk.

**Table 1:** Assumptions Involved in Constructing the Net Benefit Function

A1: Fatalities from severe malaria occur within the first few days following hospitalization. A2: Mortality differences between fluids are assumed additive on the log-odds scale. A3: Survivors and nonsurvivors receive identical treatment, except that survivors have a longer mean stay in the hospital. This additional stay is associated with an incremental cost of *C^H^*. A4: Neurological sequelae (NS) in survivors become apparent within 28 d of discharge. A5: Giving fluid resuscitation to children who would survive anyway has minimal impact on their likelihood of developing NS. A6: Improved survival from treatment is associated with a change *d^S^* in the log-odds of NS among those who are “saved” by treatment (see main text for further explanation). A7: A proportion *p^L^* of NS cases at 28 d will persist long term. A8: Short-term cases of NS have no impact on treatment costs or quality of life. A9: Survivors who are free of long-term NS have a mean quality-adjusted life expectancy of *q^M^*. A10: Long-term NS is associated with a reduction in quality-adjusted life expectancy of *Q^S^* and increased discounted lifetime management costs of *C^S^*. These values are assumed to be the same irrespective of fluids received. A11: Parameters are assumed to be independent unless correlations are induced between them through joint estimation from a common data source.

There are 4 possible outcomes for patients in the model: death, NS-free survival, survival with short-term NS, and survival with long-term NS. Table 2 lists the probability of each outcome with or without fluid resuscitation, and a full list of model parameters is given in Table 3. To assess the cost-effectiveness of treatment *j*, we calculate its incremental net benefit *B_j_*(θ) relative to no fluid, that is, the additional costs incurred and health utility gained above those observed without fluid resuscitation. This includes the cost $C_{j}^{F}$of fluid *j* and additional costs and benefits related to changes in the proportion achieving each of the outcomes in Table 2, that is,

$$\begin{array}{c} B_{j}(\theta )=\left(\frac{\exp(\alpha )}{\left(1+\exp(\alpha )\right)}-\frac{\exp(\alpha +d_{j}^{M})}{\left(1+\exp(\alpha +d_{j}^{M})\right)}\right)\\ \;(Wq^{M}-C^{H}-p^{L}\left(\frac{p^{B}\exp(d^{S})}{\left(1+p^{B}\exp(d^{S})\right)}\right)(Wq^{S}+C^{S}))\;-C_{j}^{F} \end{array}$$

where θ = $\left\{\alpha ,d_{j}^{M},q^{M},q^{S},p^{L},d^{S}\right\}$, *W* = willingness to pay per quality-adjusted life-year.

**Table 2:** Possible Outcomes for Patients in the Economic Model, with the Probability of Each Outcome and Associated Health Utility and Costs

Patient Outcome	Health Utility (QALYs)	Cost	Probability without Fluids	Probability with Fluid *j*
O1: Dies	0	$C_{j}^{F}$	$\frac{\exp(\alpha )}{\left(1+\exp(\alpha )\right)}$	$\frac{\exp\left(\alpha +d_{j}^{M}\right)}{\left(1+\exp\left(\alpha +d_{j}^{M}\right)\right)}$
O2: Survives, no NS	$q^{M}$	$C^{H}+C_{j}^{F}$	$\left(1-\frac{\exp\left(\alpha \right)}{\left(1+\exp\left(\alpha \right)\right)}\right)\left(1-p^{B}\right)$	$1-\left(\frac{\exp\left(\alpha \right)}{\left(1+\exp\left(\alpha \right)\right)}\right)\left(1-p^{B}\right)$+$\left(\frac{\exp\left(\alpha \right)}{\left(1+\exp\left(\alpha \right)\right)}-\frac{\exp\left(\alpha +d_{j}^{M}\right)}{\left(1+\exp\left(\alpha +d_{j}^{M}\right)\right)}\right)\left(1-\frac{p^{B}\exp\left(d^{S}\right)}{\left(1+p^{B}\exp\left(d^{S}\right)\right)}\right)$
O3: Survives, NS at 28 d but not long-term	$q^{M}$	$C^{H}+C_{j}^{F}$	$\left(1-\frac{\exp\left(\alpha \right)}{\left(1+\exp\left(\alpha \right)\right)}\right)\left(1-p^{L}\right)p^{B}$	$1-\left(\frac{\exp\left(\alpha \right)}{\left(1+\exp\left(\alpha \right)\right)}\right)\left(1-p^{L}\right)p^{B}$+$\left(\frac{\exp\left(\alpha \right)}{\left(1+\exp\left(\alpha \right)\right)}-\frac{\exp\left(\alpha +d_{j}^{M}\right)}{\left(1+\exp\left(\alpha +d_{j}^{M}\right)\right)}\right)\left(\frac{p^{B}\exp\left(d^{S}\right)}{\left(1+p^{B}\exp\left(d^{S}\right)\right)}\right)\left(1-p^{L}\right)$
O4: Survives with long-term NS	$q^{M}-q^{S}$	$C^{H}+C^{S}+C_{j}^{F}$	$\left(1-\frac{\exp\left(\alpha \right)}{\left(1+\exp\left(\alpha \right)\right)}\right)p^{L}p^{B}$	$1-\left(\frac{\exp\left(\alpha \right)}{\left(1+\exp\left(\alpha \right)\right)}\right)p^{L}p^{B}$+$\left(\frac{\exp\left(\alpha \right)}{\left(1+\exp\left(\alpha \right)\right)}-\frac{\exp\left(\alpha +d_{j}^{M}\right)}{\left(1+\exp\left(\alpha +d_{j}^{M}\right)\right)}\right)\left(\frac{p^{B}\exp\left(d^{S}\right)}{\left(1+p^{B}\exp\left(d^{S}\right)\right)}\right)p^{L}$

Note: NS = neurological sequelae; QALY = quality-adjusted life-year.

**Table 3:** Values/Distributions Used for Parameters in the Economic Model and Study Designs That Would Provide Further Information on Them

Parameter	Description	Value/Distribution	Study Design to Provide Further Information
$d_{2}^{M}$	Effect of albumin on mortality	Posterior distribution generated by Bayesian evidence synthesis model	RCT including albumin and control arm
$d_{3}^{M}$	Effect of saline on mortality	Posterior distribution generated by Bayesian evidence synthesis model	RCT including saline and control arm
$d_{4}^{M}$	Effect of gelofusine on mortality	Posterior distribution generated by Bayesian evidence synthesis model	RCT including gelofusine and control arm
d^S^	Change to NS risk in “saved” patients	Posterior distribution generated by Bayesian evidence synthesis model	RCT including fluid resuscitation arm(s) and control arm
$C^{F}$	Fluid cost (per patient)	$1 (saline), $35 (albumin), $12.50 (gelofusine)	
$C^{H}$	Additional in-patient costs associated with survival	$60 (based on 5 d at $12/d)	
α	Log-odds of death without fluid resuscitation	Normal with implied median probability of death 25%, 95% CI 15%–40%	Cohort study with short-term follow-up
$p^{B}$	Probability of NS without fluid resuscitation	Beta (1,9) (mean 10%, 95% CI 0.6%–28.5%)	Cohort study with 28-d follow-up
$p^{L}$	Probability that NS will still be present at 6 mo conditional on NS observed at 28 d	Beta (1,1)	Cohort study with 6-mo follow-up
$C^{S}$	Long-term discounted costs of NS	$20 000	
${\text{q}}^{M}$	QALY loss per fatality	Normal with CHAR1 = 20 and *s* = 5	Cohort study on survivors without NS: long-term follow-up
${\text{q}}^{\text{S}}$	QALY loss per case of NS	Truncated normal with CHAR1 = 5, *s* = 3.16, lower limit = 0	Cohort study on those with NS: long-term follow-up

Note: CI = confidence interval; NS = neurological sequelae; QALY = quality-adjusted life-year; RCT = randomized controlled trial.

This model (eq. 1) was designed to estimate the cost-effectiveness of fluids once FEAST trial results were available. However, it can also be used in an EVPPI analysis to provide insight on the potential value of the FEAST trial and other studies that could be undertaken to inform parameters in eq. 1. This requires quantifying the uncertainty around the parameters implied by information available at the time the FEAST trial was designed (Table 3). Values for $C^{H}$, $C^{F}$, and $q^{M}$were based on health economic analysis carried out by several of the authors alongside the FEAST trial.^16^ Data on NS-related economic parameters are sparse; our chosen values are toward the upper end of what were considered plausible values, for reasons that are explored in the Discussion section.

Table 3 also lists the type of future studies that can provide information on each of the uncertain model parameters. We assumed that baseline mortality in a randomized controlled trial (RCT) population would be different from that in the decision populations, so that a different study type would be needed to provide information on baseline and relative effect parameters. In practice, if the 2 populations are considered sufficiently similar, all of these parameters could be estimated from an RCT. Below, we present generic methods that can be used to estimate EVPPI in a wide range of circumstances. We then illustrate their application for the fluid resuscitation example by considering the different future studies that we could run (Table 3) and the subset of parameters those studies would directly inform.

## Methods

Let $B_{j}(\theta )$be a general form for the incremental net benefit of intervention *j* given model parameters θ and j* be the optimal decision given current information. We define ϕ as a subset of (focal) parameters on which we are considering collecting further information on in a new study, and ϕ^*C*^ as the set of remaining (nonfocal) model parameters. EVPPI(φ) equals the expected gain in net benefit, given current information, from switching to the optimum treatment once ϕ is known with certainty:

$$\mathrm{EVPPI}\left(\text{φ}\right)=\underset{\text{φ}}{E}\left[\underset{j}{\max}\left\{\underset{{\text{φ}}^{C}|\text{φ}}{E}\left[B_{j}(\theta )-B_{j*}(\theta )\right]\right\}\right].$$

We describe 2 types of methods that can be used (if necessary in combination) to avoid the inner-simulation step when calculating EVPPI(φ), which all involve finding a solution (exact or approximate) for the inner expectation$\underset{{\text{φ}}^{C}|\text{φ}}{E}\left[B_{j}(\theta )-B_{j*}(\theta )\right]$.

### Methods in Which Expectations for (Functions of) φ^c^ Can Be Plugged in Directly

#### Method 1: Direct Substitution of Means of the Nonfocal Parameters in a Linear Net Benefit Function

This method is applicable when the net benefit takes the form

$$B_{j}({\text{φ}}^{C},\text{φ})=\sum_{i}f_{i,j}\left(\text{φ}\right){\text{φ}}_{i}^{C},$$

where ${\text{φ}}_{i}^{C}$,the *i*th component of ${\text{φ}}^{C}$, is independent of any of the elements of φ in its linear coefficient $f_{i,j}\left(\text{φ}\right)$. Given this independence, it has been shown^4,9^ that

$$\underset{{\text{φ}}^{C}|\text{φ}}{E}\left[B_{j}({\text{φ}}^{C},\text{φ})\right]=\sum_{i}f_{i,j}\left(\text{φ}\right)E\left[{\text{φ}}_{i}^{C}\right].$$

EVPPI(φ)can now be estimated in a single Monte Carlo simulation by substituting equation 3 into equation 2.

#### Method 2: Direct Substitution of Means of the Nonfocal Parameters in a Multilinear Net Benefit Function

This method is applicable when the net benefit can be expressed in the form

$$B_{j}({\text{φ}}^{C},\text{φ})=\sum_{i}f_{i,j}\left(\text{φ}\right)g_{i,j}\left({\text{φ}}^{C}\right),$$

where

- each function $g_{i,j}\left({\text{φ}}^{C}\right)$ is a product of mutually independent elements of ${\text{φ}}^{C}$ and
- each of the elements of $g_{i,j}\left({\text{φ}}^{C}\right)$ is independent of the elements of φ in its linear coefficient $f_{i,j}\left(j,\text{φ}\right)$.

If these conditions are satisfied, it has been shown^4,9^ that the expected value of each function $g_{i,j}\left({\text{φ}}^{C}\right)$will equal the product of the expectations of its components, so that

$$\underset{{\text{φ}}^{C}|\text{φ}}{E}\left[B_{j}({\text{φ}}^{C},\text{φ})\right]=\sum_{i}f_{i,j}\left(\text{φ}\right)g_{i,j}\left(E\left({\text{φ}}_{1}^{C}\right),E\left({\text{φ}}_{2}^{C}\right),\ldots \right).$$

EVPPI(φ)can now be estimated in a single Monte Carlo simulation by substituting equation 4 into equation 2.

#### Method 3: Reparameterization to Linearize the Net Benefit Function

This method is applicable when the net benefit can be expressed in the form

$$B_{j}({\text{φ}}^{C},\text{φ})=\sum_{i}f_{i,j}(\text{φ})\beta_{i,j}\left({\text{φ}}^{C}\right)$$

where

- $\beta_{i,j}\left({\text{φ}}^{C}\right)$ are any functions of the ${\text{φ}}^{C}$ parameters and
- the elements of ${\text{φ}}^{C}$ in each $\beta_{i,j}\left({\text{φ}}^{C}\right)$are all independent of any of the elements of φ in its linear coefficient $f_{i,j}(\text{φ})$.

Method 2 is not applicable if any of the $\beta_{i,j}\left({\text{φ}}^{C}\right)$are not products of their components and/or include correlated nonfocal parameters . However, the second condition ensures the independence of each $\beta_{i,j}\left({\text{φ}}^{C}\right)$ from its linear coefficient $f_{i,j}(\text{φ})$, which implies that

$$\underset{{\text{φ}}^{C}|\text{φ}}{E}\left[B_{j}({\text{φ}}^{C},\text{φ})\right]=f_{i,j}(\text{φ})\underset{{\text{φ}}^{C}}{E}\left[\beta_{i,j}\left({\text{φ}}^{C}\right)\right].$$

EVPPI(φ)can now be estimated in a single Monte Carlo simulation by substituting equation 5 into equation 2. This method has been used by Welton and others.^17^

### Methods Involving Functions Approximating the Conditional Expectations of ${\text{φ}}^{C}$

#### Method 4: Taylor Series Approximations to the Net-Benefit Function

This method is applicable when net benefit takes the form

$$B_{j}({\text{φ}}^{C},\text{φ})=\sum_{i}f_{i,j}(\text{φ})\beta_{i,j}\left({\text{φ}}_{-i}^{C}\right)h_{i,j}\left({\text{φ}}_{i}^{C},\text{φ}\right),$$

where

- $f_{i,j}(\text{φ})$are arbitrary functions of the focal parameters and $h_{i,j}\left({\text{φ}}_{i}^{C},\text{φ}\right)$are arbitrary smooth nonlinear functions of φ and a single element ${\text{φ}}_{i}^{C}$ of ${\text{φ}}^{C}$,
- $\beta_{i,j}\left({\text{φ}}_{-i}^{C}\right)$are arbitrary functions of the remaining ${\text{φ}}^{C}$ parameters (excluding ${\text{φ}}_{i}^{C}$), and
- ${\text{φ}}_{i}^{C}$, the elements of φ in $h_{i,j}\left({\text{φ}}_{i}^{C},\text{φ}\right)$, and the elements of ${\text{φ}}^{C}$in $\beta_{i,j}\left({\text{φ}}_{-i}^{C}\right)$ are mutually independent.

Because ${\text{φ}}_{i}^{C}$, ${\text{φ}}_{-i}^{C}$ and the elements of φ *in $h_{i,j}\left({\text{φ}}_{i}^{C},\text{φ}\right)$* are mutually independent, we can decompose the expectation:

$$\underset{{\text{φ}}^{C}|\text{φ}}{E}\left[\sum_{i}f_{i,j}(\text{φ})\beta_{i,j}\left({\text{φ}}_{-i}^{C}\right)h_{i,j}\left({\text{φ}}_{i}^{C},\text{φ}\right)\right]=\sum_{i}f_{i,j}(\text{φ})E\left[\beta_{i,j}\left({\text{φ}}_{-i}^{C}\right)\right]E\left[h_{i,j}\left({\text{φ}}_{i}^{C},\text{φ}\right)\right].$$

The expectation of each $\beta_{i,j}\left({\text{φ}}_{-i}^{C}\right)$, if required, can be found using method 3. Because $h_{i,j}\left({\text{φ}}_{i}^{C},\text{φ}\right)$is nonlinear, its expectation conditional on φ will not equal $h_{i,j}\left(E\left({\text{φ}}_{i}^{C}\right),\text{φ}\right)$, and we cannot use methods 1 to 3. Our strategy is instead to construct a function of $E\left({\text{φ}}_{i}^{C}\right)$ that approximates the required conditional expectation and so can be used to replace the inner simulation step. One approach is to use Taylor series expansions.^9^ The *n*th-order Taylor series expansion of $h_{i,j}\left({\text{φ}}_{i}^{C},\text{φ}\right)$ in the neighborhood of $E\left({\text{φ}}_{i}^{C}\right)$ is given by

$$h_{i,j}\left({\text{φ}}_{i}^{C},\text{φ}\right)≃h_{i,j}(E[{\text{φ}}_{i}^{C}],\text{φ})+\sum_{r=1}^{n}\frac{1}{r!}\frac{d^{r}h_{i,j}\left(E[{\text{φ}}_{i}^{C}],\text{φ}\right)}{d{\left({\text{φ}}_{i}^{C}\right)}^{r}}{\left({\text{φ}}_{i}^{C}-E[{\text{φ}}_{i}^{C}]\right)}^{r}.$$

As long as ${\text{φ}}_{i}^{C}$ is independent of the elements of φ in $h_{i,j}\left({\text{φ}}_{i}^{C},\text{φ}\right)$, taking conditional expectations on both sides gives

$$\underset{{\text{φ}}_{i}^{C}|\text{φ}}{E}\left[h_{i,j}\left({\text{φ}}_{i}^{C},\text{φ}\right)\right]≃h_{i,j}(E[{\text{φ}}_{i}^{C}],\text{φ})+\frac{1}{2}\frac{d^{2}h_{i,j}\left(E[{\text{φ}}_{i}^{C}],\text{φ}\right)}{d{\left({\text{φ}}_{i}^{C}\right)}^{2}}\cdot \mathrm{Var}[{\text{φ}}_{i}^{C}]+\ldots$$

This can be used to give an approximation to the conditional expectation of the net benefit function; for example, a second-order approximation is

$$\underset{{\text{φ}}^{C}|\text{φ}}{E}\left[B_{j}({\text{φ}}^{C},\text{φ})\right]\approx \sum_{i}f_{i,j}\left(\text{φ}\right){\bar{h}}_{i,j}^{T2}(E\left[{\text{φ}}_{i}^{C}\right],\text{φ})E\left[\beta_{i,j}\left({\text{φ}}_{-i}^{C}\right)\right],$$

where

$${\bar{h}}_{i,j}^{T2}(E\left[{\text{φ}}_{i}^{C}\right],\text{φ})=h_{i,j}(E[{\text{φ}}_{i}^{C}],\text{φ})+\frac{1}{2}\frac{d^{2}h_{i,j}\left(E[{\text{φ}}_{i}^{C}],\text{φ}\right)}{d{\left({\text{φ}}_{i}^{C}\right)}^{2}}\cdot \mathrm{Var}[{\text{φ}}_{i}^{C}].$$

EVPPI(φ)can now be estimated in a single Monte Carlo simulation by substituting equation 6 into equation 2.

The form of the approximation will depend on the functional form of $h_{i,j}\left({\text{φ}}_{i}^{C},\text{φ}\right)$. For example, net benefit functions commonly consist of an absolute probability that is informed by parameters on the log-odds scale (baseline log-odds plus a log-odds ratio for treatment *j*), so that $h_{i,j}\left({\text{φ}}_{i}^{C},\text{φ}\right)$is the inverse-logit function of the form

$$h_{i,j}({{\text{φ}}_{i}}^{C},\text{φ})=\frac{e^{\left({{\text{φ}}_{i}}^{C}+\text{φ}\right)}}{1+e^{\left({{\text{φ}}_{i}}^{C}+\text{φ}\right)}}.$$

The second-order Taylor series approximation for the inverse-logit function is (see supplementary appendix):

$$\begin{array}{c} \underset{{\text{φ}}^{C}|\text{φ}}{E}\left[h(j,{\text{φ}}_{i}^{C},\text{φ})\right]≃H+\frac{H(1-H)(1-2H)\mathrm{Var}[{\text{φ}}_{i}^{C}]}{2}\\ \text{where}\;H=\frac{e^{\left(E\left[{\text{φ}}_{i}^{C}\right]+\text{φ}\right)}}{1+e^{\left(E\left[{\text{φ}}_{i}^{C}\right]+\text{φ}\right)}}. \end{array}$$

Occasionally, the absolute probability is informed by parameters on the cumulative log-log (clog-log) scale, so that function *h* is the inverse-clog-log function of the form

$$h_{i,j}({{\text{φ}}_{i}}^{C},\text{φ})=1-e^{-e^{\left({{\text{φ}}_{i}}^{C}+\text{φ}\right)}}.$$

In this case, it can be shown that the second-order Taylor approximation gives (see supplementary appendix)

$$\begin{array}{c} \underset{{\text{φ}}^{C}|\text{φ}}{E}\left[h(j,{\text{φ}}_{i}^{C},\text{φ})\right]≃1-H+\frac{H\ln\left(H\right)\left(\ln\left(H\right)-1\right)\mathrm{Var}[{\text{φ}}_{i}^{C}]}{2}\\ \text{where}\;H=e^{-e^{\left(E\left[{\text{φ}}_{i}^{C}\right]+\text{φ}\right)}} \end{array}.$$

**Figure 1:**
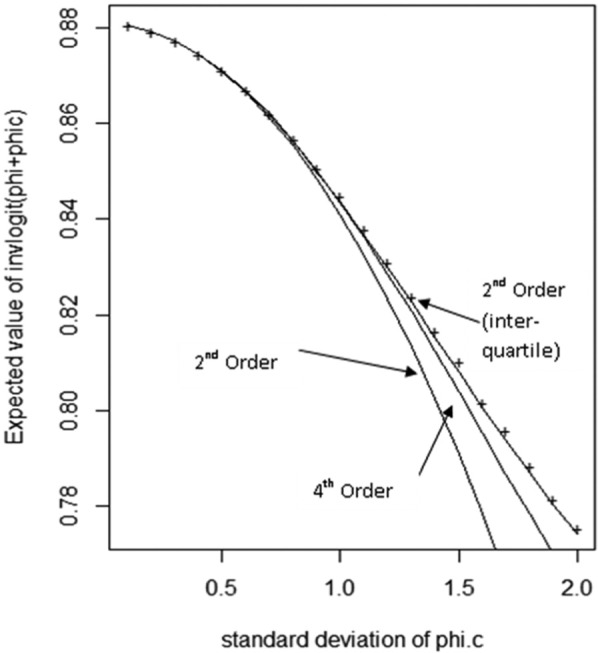
Taylor series approximations for the expectation of the inverse logit of ($\text{φ}+{\text{φ}}_{i}^{C}$), where φ is known, ${\text{φ}}_{i}^{C}$is normally distributed, and E[$\text{φ}+{\text{φ}}_{i}^{C}$] = 2. Pluses mark true values for the expectation as the standard deviation of ${\text{φ}}_{i}^{C}$ varies, and approximations are based around a single expansion around the mean (second- and fourth-order approximation) and averaging over approximations derived at each interquartile mean (second-order approximation).

When using Taylor series approximations, it is important to assess the accuracy of the approximation and to consider adaptations that improve accuracy, for example, the addition of higher-order terms. We have developed the following adaptation for this purpose. We split the prior distribution for ${\text{φ}}_{i}^{C}$ into quantiles, Q, and find the mean, $\mu_{Q}$, of ${\text{φ}}_{i}^{C}$ conditional on lying in quantile Q. We then construct separate Taylor series approximations to $h_{i,j}({{\text{φ}}_{i}}^{C},\text{φ})$ within each quantile around the quantile-specific mean, $\mu_{Q}$. It can be shown that the expected value of the average over this set of Taylor series approximations converges to the required quantity, $\underset{{\text{φ}}^{C}|\text{φ}}{E}\left[h(j,{\text{φ}}_{i}^{C},\text{φ})\right]$ (details and proof in the supplementary appendix). Figure 1 illustrates the accuracy of Taylor series expansions in the case in which ${{\text{φ}}_{i}}^{C}$ is normally distributed, $h_{i,j}({{\text{φ}}_{i}}^{C},\text{φ})$ takes the form given in equation 6, and E[$\text{φ}+{\text{φ}}_{i}^{C}$] = 2. The accuracy of approximations based on Taylor series expansions around E[${\text{φ}}_{i}^{C}$] reduces as the variance of ${\text{φ}}_{i}^{C}$ increases, and the fourth-order approximation is noticeably more accurate than the second-order approximation. However, averaging over second-order expansions around the interquartile means is more effective in improving accuracy than increasing the order of the single approximation. The Taylor series gives local approximations whose accuracy diminishes as the distance from the expansion point increases. Therefore, averaging over multiple expansions around a range of values reduces error in the approximation by restricting the range over which each approximation is made, and this is our recommended approach.

#### Method 5: Spline-Approximation Methods When There Are Correlations between Parameters

This method is applicable when the net benefit takes the form

$$B_{j}({\text{φ}}^{C},\text{φ})=\sum_{i}f_{i,j}(\text{φ})h_{i,j}\left({\text{φ}}^{C},{\text{φ}}_{i}\right),$$

where

- $f_{i,j}(\text{φ})$ are arbitrary functions of the focal parameters,
- $h_{i,j}\left({\text{φ}}^{C},{\text{φ}}_{i}\right)$ are arbitrary smooth nonlinear functions of ${\text{φ}}^{C}$ and a single element ${\text{φ}}_{i}$ of φ, and
- for at least one value of i, ${\text{φ}}_{i}$ is correlated with one or more of the elements of ${\text{φ}}^{C}$ in $h_{i,j}\left({\text{φ}}^{C},{\text{φ}}_{i}\right)$.

Here we can no longer use Taylor series expansions to estimate the conditional expectation of $h_{i,j}\left({\text{φ}}^{C},{\text{φ}}_{i}\right)$ in terms of the unconditional expectation of ${\text{φ}}^{C}$, because elements of the latter are correlated with${\text{φ}}_{i}$. Instead, we aim to construct functions $\xi_{i,j}\left({\text{φ}}_{i}\right)$ whose values are close approximations to $\underset{{\text{φ}}^{C}|\text{φ}}{E}\left[h_{i,j}({\text{φ}}_{i}^{C},\text{φ})\right]$, so that

$$\underset{{\text{φ}}^{C}|\text{φ}}{E}\left[B_{j}({\text{φ}}^{C},\text{φ})\right]\approx \sum_{i}f_{i,j}\left(\text{φ}\right)\xi_{i,j}\left({\text{φ}}_{i}\right).$$

EVPPI(φ) can then be estimated in a single Monte Carlo simulation by substituting equation 8 into equation 2.

An obvious choice for $\xi_{i,j}\left({\text{φ}}_{i}\right)$ is a spline.^18^ There are several different types of splines; we describe here the use of restricted cubic splines for this purpose. The restricted cubic spline $\xi_{i,j}\left({\text{φ}}_{i}\right)$ takes the form

$$\xi_{i,j}\left({\text{φ}}_{i}\right)=\gamma_{j,0}+\gamma_{j,1}+\Sigma_{n=1}^{N_{\tau }}\gamma_{j,n+1}{\left(\max\;\left(0,{\text{φ}}_{i}-\tau_{n}\right)\right)}^{3},$$

where $\left\{\tau_{1},\ldots \tau_{N_{\tau }}\right\}$ are the N_τ_ knots that define the breakpoints of $\xi_{i,j}\left({\text{φ}}_{i}\right)$. The parameters γ are chosen so that $\xi_{i,j}\left({\text{φ}}_{i}\right)$ is linear for values of ${\text{φ}}_{i}$ less than τ_1_ or greater than τ_Nτ_. This requires a minimum of 3 knots. Furthermore, $\xi_{i,j}\left({\text{φ}}_{i}\right)$ must be continuously differentiable, which places further restrictions on the γ. To construct these splines, we first require estimates of $\underset{{\text{φ}}^{C}|\text{φ}}{E}\left[h_{i,j}({\text{φ}}^{C},{\text{φ}}_{i})\right]$ for a large set of values for ${\text{φ}}_{i}$ spanning a plausible range. Once knots $\left\{\tau_{1},\ldots \tau_{N_{\tau }}\right\}$ have been chosen, regression analysis can be used to derive coefficients γ defining the splines that fit the estimated conditional expectations most closely. This approach can be implemented by the following algorithm.

Step 1: Generate *N*_θ_ samples from the joint distribution of θ and estimate $h_{i,j}({\text{φ}}^{C},\text{φ})$ for each sample and each intervention *j*. These should be the same set of samples that will subsequently be used to estimate EVPPI(φ).
Step 2: Divide the range spanning the values of ${\text{φ}}_{i}$ into *M^B^* bins $\Phi_{m}=\{x_{m-1},x_{m}\},2\leq m\leq M^{B}$. We chose values for $x_{m}$ so that there were an equal number of samples in each bin (this is not the only possible approach, e.g., bins could be of equal width).
Step 3: For each bin $\Phi_{m}$ :
  - Identify the subset of samples within θ for which the sampled value of ${\text{φ}}_{i}$ lies within $\Phi_{m}$.
  - Calculate the mean ${\bar{\text{φ}}}_{i,m}$ of all the sampled values of ${\text{φ}}_{i}$ in the subset.
  - Calculate the mean ${\bar{h}}_{i,j,m}$ of all the sampled values of $h_{i,j}({\text{φ}}^{C},{\text{φ}}_{i})$ in the subset.
Step 4: Choose a vector of knots $\left\{\tau_{1},\ldots \tau_{N_{\tau }}\right\}$ that lie within the range spanning the values of ${\text{φ}}_{i}$ i.e., ($x_{1},x_{M^{B}}$). We chose to use equally spaced knots.
Step 5: For each intervention *j* and function *i*, we fit a restricted cubic spline regression model to estimate the spline function $\xi_{i,j}\left({\text{φ}}_{i}\right)$, based on the M^B^ pairs of values $\left\{{\bar{\text{φ}}}_{i,m},{\bar{h}}_{i,j,m}\right\}$, where ${\bar{h}}_{i,j,m}$ is the dependent variable, ${\bar{\text{φ}}}_{i,m}$ the explanatory variable, and the knots are as chosen in step 4. The estimated spline function $\xi_{i,j}\left({\text{φ}}_{i}\right)$ provides an approximation to $\underset{{\text{φ}}^{C}|{\bar{\text{φ}}}_{i}}{E}\left[h_{i,j}({\text{φ}}^{C},{\text{φ}}_{i})\right]$ that converges to the true expectation as N_θ_ and M^B^ increase. Restricted cubic splines can be fitted in many statistical packages;we used theRpackage rms (available at http://biostat.mc.vanderbilt.edu/wiki/Main/Rrms).

### EVPPI Calculations for the Fluid Resuscitation Example

We describe below, for the fluid resuscitation example (eq. 1), how EVPPI can be calculated for various different subsets of parameters, representing different potential future studies (Table 3), using the appropriate method described in the Methods section. We assume that we have a set of simulated samples from the joint prior distribution for all of the uncertain parameters.

#### Example 1: Net Benefit Linear in Nonfocal Parameters

Suppose we wish to evaluate the value of running 2 studies: 1) a 4-arm RCT (such as FEAST) to provide information on relative effects, ${\text{d}}^{\text{M}},{\text{d}}^{\text{S}}$, and 2) a cohort study with 6-mo follow-up to provide information on $\alpha ,{\text{p}}^{\text{B}},{\text{p}}^{\text{L}}$. In this case, the focal parameters (i.e., those for which we wish to calculate the EVPPI) are $\text{φ}=\left\{{\text{d}}^{\text{M}},{\text{d}}^{\text{S}},\alpha ,{\text{p}}^{\text{B}},{\text{p}}^{\text{L}}\right\}$, so that ${\text{φ}}^{C}=\left\{{\text{q}}^{\text{M}},{\text{q}}^{\text{S}}\right\}$. By defining

$$f_{1,j}(\text{φ})=W\left(\frac{\exp(\alpha )}{\left(1+\exp(\alpha )\right)}-\frac{\exp(\alpha +d_{j}^{M})}{\left(1+\exp(\alpha +d_{j}^{M})\right)}\right)$$

$$f_{2,j}(\text{φ})=p^{L}W\left(\frac{\exp(\alpha )}{\left(1+\exp(\alpha )\right)}-\frac{\exp(\alpha +d_{j}^{M})}{\left(1+\exp(\alpha +d_{j}^{M})\right)}\right)\left(\frac{p^{B}\exp(d^{S})}{\left(1+p^{B}\exp(d^{S})\right)}\right)$$

$$f_{3,j}(\text{φ})=\left(\frac{\exp(\alpha )}{\left(1+\exp(\alpha )\right)}-\frac{\exp(\alpha +d_{j}^{M})}{\left(1+\exp(\alpha +d_{j}^{M})\right)}\right)\left(C^{H}+p^{L}C^{S}\frac{p^{B}\exp(d^{S})}{\left(1+p^{B}\exp(d^{S})\right)}\right)+C_{j}^{F},$$

the net benefit can be expressed as

$$B_{j}({\text{φ}}^{C},\text{φ})=f_{1,j}\left(\text{φ}\right)q^{M}-f_{2,j}\left(\text{φ}\right)q^{S}-f_{3,j}\left(\text{φ}\right).$$

This satisfies the conditions that must be met to use method 1 for single-step Monte Carlo estimation of EVPPI. Therefore, we can apply equations 1 and 2 to restate EVPPI(φ) in a form requiring only a single simulation step:

$$\begin{array}{c} \mathrm{EVPPI}\left(\text{φ}\right)=\mathrm{EVPPI}\left({\text{d}}^{\text{M}},\alpha ,{\text{p}}^{\text{B}},{\text{d}}^{\text{S}},{\text{p}}^{\text{L}}\right)\\ \;=\underset{\text{φ}}{E}\left[\underset{j}{\max}\left\{B_{j}(\text{φ},E[q^{M}],E[{\text{q}}^{\text{S}}])-B_{j*}(\text{φ},E[q^{M}],E[{\text{q}}^{\text{S}}])\right\}\right] \end{array}.$$

Method 1 can also be used when ${\text{φ}}^{C}=\left\{{\text{q}}^{\text{M}},{\text{p}}^{\text{L}}\right\}$ or when ${\text{φ}}^{C}$ equals any single element of $\left\{{\text{q}}^{\text{M}},{\text{q}}^{S},{\text{p}}^{\text{L}}\right\}$.

#### Example 2: Net Benefit Multilinear in Nonfocal Parameters

Suppose we wish to evaluate the value of running 3 studies: 1) a 4-arm RCT (such as FEAST) to provide information on relative effects, ${\text{d}}^{\text{M}},{\text{d}}^{\text{S}}$, 2) a cohort study with 28-d follow-up (or routine data) to provide information on $\alpha ,{\text{p}}^{\text{B}}$, and 3) a long-term cohort study on patients who survive without NS to provide information on ${\text{q}}^{M}$. In this case,

$\text{φ}=\left\{{\text{d}}^{\text{M}},\alpha ,{\text{p}}^{\text{B}},{\text{d}}^{\text{S}},{\text{q}}^{M}\right\}$, so that ${\text{φ}}^{C}=\left\{p^{L},{\text{q}}^{\text{S}}\right\}$. Defining

$$f_{1,j}(\text{φ})=W\left(\frac{\exp(\alpha )}{\left(1+\exp(\alpha )\right)}-\frac{\exp(\alpha +d_{j}^{M})}{\left(1+\exp(\alpha +d_{j}^{M})\right)}\right)\left(\frac{p^{B}\exp(d^{S})}{\left(1+p^{B}\exp(d^{S})\right)}\right)$$

$$f_{2,j}(\text{φ})=C^{S}\left(\frac{\exp(\alpha )}{\left(1+\exp(\alpha )\right)}-\frac{\exp(\alpha +d_{j}^{M})}{\left(1+\exp(\alpha +d_{j}^{M})\right)}\right)\left(\frac{p^{B}\exp(d^{S})}{\left(1+p^{B}\exp(d^{S})\right)}\right)$$

$$f_{3,j}(\text{φ})=\left(\frac{\exp(\alpha )}{\left(1+\exp(\alpha )\right)}-\frac{\exp(\alpha +d_{j}^{M})}{\left(1+\exp(\alpha +d_{j}^{M})\right)}\right)\left(Wq^{M}-C^{H}\right)-C_{j}^{F}$$

net benefit can be expressed as

$$B(j,{\text{φ}}^{C},\text{φ})=-f_{1,j}\left(\text{φ}\right)p^{L}q^{S}-f_{2,j}\left(\text{φ}\right)p^{L}+f_{3,j}\left(\text{φ}\right)$$

This satisfies the conditions that must be met to use method 2 for single-step Monte Carlo estimation of EVPPI. Therefore, we can apply equations 2 and 4 to restate EVPPI(φ) in a form requiring only a single simulation step:

$$\begin{array}{c} \mathrm{EVPPI}\left(\text{φ}\right)=\mathrm{EVPPI}\left({\text{d}}^{\text{M}},\alpha ,{\text{p}}^{\text{B}},{\text{d}}^{\text{S}},{\text{q}}^{\text{M}}\right)\\ \;=\underset{\text{φ}}{E}\left[\underset{j}{\max}\left\{B_{j}(\text{φ},E[{\text{p}}^{\text{L}}],E[{\text{q}}^{\text{S}}])-B_{j*}(\text{φ},E[{\text{p}}^{\text{L}}],E[{\text{q}}^{\text{S}}])\right\}\right] \end{array}.$$

Method 2 can also be used when ${\text{φ}}^{C}=\left\{{\text{q}}^{\text{M}},{\text{q}}^{S},{\text{p}}^{\text{L}}\right\}$.

#### Example 3: Net-Benefit Linear in Functions of the Nonfocal Parameters

Suppose we wish to assess the value of running a cohort study with long-term follow-up, including patients with and without NS, to estimate persistence rates and long-term quality of life.

Here $\text{φ}=\left\{{\text{q}}^{\text{M}},{\text{q}}^{S},{\text{p}}^{\text{L}}\right\}$, so that ${\text{φ}}^{C}=\left\{{\text{d}}^{\text{M}},\alpha ,{\text{p}}^{\text{B}},{\text{d}}^{\text{S}}\right\}$. The net benefit function is no longer multilinear in ${\text{φ}}^{C}$, so neither of methods 1 or 2 is valid. However, by defining

$$\beta_{1,j}\left({\text{φ}}^{C}\right)=\frac{\exp(\alpha )}{\left(1+\exp(\alpha )\right)}-\frac{\exp(\alpha +d_{j}^{M})}{\left(1+\exp(\alpha +d_{j}^{M})\right)}$$

$$\beta_{2,j}\left({\text{φ}}^{C}\right)=\left(\frac{\exp(\alpha )}{\left(1+\exp(\alpha )\right)}-\frac{\exp(\alpha +d_{j}^{M})}{\left(1+\exp(\alpha +d_{j}^{M})\right)}\right)\left(\frac{p^{B}\exp(d^{S})}{\left(1+p^{B}\exp(d^{S})\right)}\right)$$

$$f_{1}\left(\text{φ}\right)=Wq^{M}-C^{H}$$

$$f_{2}\left(\text{φ}\right)=p^{L}\left(Wq^{S}+C^{S}\right),$$

the net benefit can be expressed as

$$B_{j}({\text{φ}}^{C},\text{φ})=f_{1}\left(\text{φ}\right)\beta_{1,j}\left({\text{φ}}^{C}\right)-f_{2}\left(\text{φ}\right)\beta_{2,j}\left({\text{φ}}^{C}\right)-C_{j}^{F}.$$

This satisfies the conditions that must be met to use method 3 for single-step Monte Carlo estimation of EVPPI. Therefore, once the unconditional expectations $E\left[\beta_{1,j}\left({\text{φ}}^{C}\right)\right]$ and $E\left[\beta_{2,j}\left({\text{φ}}^{C}\right)\right]$ are known, we can apply equations 2 and 5 to restate EVPPI(φ) in a form requiring only a single simulation step:

$$EVPPI\left(\varphi \right)=EVPPI\left({\text{q}}^{\text{M}},{\text{q}}^{\text{S}},{\text{p}}^{\text{L}}\right)\underset{\varphi }{=E}\left[\underset{j}{\max}\left\{B_{j}\left(\varphi ,E\left[\beta_{1,j}\left(\varphi^{C}\right)\right],E\left[\beta_{2,j}\left(\varphi^{C}\right)\right]\right)-B_{j*}\left(\varphi ,E\left[\beta_{1,j}\left(\varphi^{C}\right)\right],E\left[\beta_{2,j}\left(\varphi^{C}\right)\right]\right)\right\}\right].$$

Method 3 can also be used when φ is any subset of $\left\{{\text{q}}^{\text{M}},{\text{q}}^{S},{\text{p}}^{\text{L}}\right\}$.

#### Example 4: Net Benefit Includes Joint Nonlinear Functions of Independent Focal and Nonfocal Parameters

Suppose we wish to evaluate the value of running a 4-arm RCT (such as FEAST) to provide information on relative effects, ${\text{d}}^{\text{M}},{\text{d}}^{\text{S}}$. Here, $\text{φ}=\{{\text{d}}^{\text{M}},{\text{d}}^{\text{S}}\}$, so that ${\text{φ}}^{C}=\left\{\alpha ,{\text{p}}^{\text{B}},p^{L},{\text{q}}^{\text{S}},{\text{q}}^{M}\right\}$. In this case, by defining

$$h_{1,j}(\alpha ,\text{φ})=\frac{\exp(\alpha +d_{j}^{M})}{\left(1+\exp(\alpha +d_{j}^{M})\right)}$$

$$h_{2}(p^{B},\text{φ})=\left(\frac{\exp(d^{S}+\ln(p^{B}))}{\left(1+\exp(d^{S}+\ln\left(p^{B}\right)\right)}\right)$$

$$\beta_{1}(q^{M})=\left(Wq^{M}-C^{H}\right)$$

$$\beta_{2}(q^{M})=p^{L}\left(Wq^{S}+C^{S}\right),$$

the net benefit can be expressed as

$$B_{j}\left({\text{φ}}^{C},\text{φ}\right)=\left(h_{1,j}\left(\alpha ,\text{φ}\right)-h_{1,1}\left(\alpha ,\text{φ}\right)\right)\left(\beta_{1}\left(q^{M}\right)-h_{2}\left(p^{B},\text{φ}\right)\beta_{2}\left({\text{φ}}^{C}\right)\right)-C_{j}^{F}.$$

Because α and *p^B^* are independent and *d^M^* and *d^S^* are known, the terms h_1_, h_2_, β_1_, and β_2_ are mutually independent. Therefore, we can write

$$\begin{array}{c} \mathrm{EVPPI}\left[{\text{d}}^{\text{M}},{\text{d}}^{\text{S}}\right]=\underset{\text{φ}}{E}\left[\underset{j}{\max}\left\{{B\prime }_{j}-{B\prime }_{j*}\right\}\right]\\ \text{where}\\ {B\prime }_{j}=B_{j}\left(\text{φ},\beta_{1}\left(E\left[q^{M}\right]\right),\beta_{2}\left(E\left[p^{L}\right],E\left[q^{S}\right]\right),E\left[h_{1,1}\left(\alpha \right)\right],E\left[h_{1,j}\left(\text{φ},\alpha \right)\right],E\left[h_{2}\left(\text{φ},p^{B}\right)\right]\right) \end{array}.$$

$E\left[h_{1,j}\left(\text{φ},\alpha \right)\right]$ (a function of of α and $d_{j}^{M}$) and $E\left[h_{2}\left(\text{φ},p^{B}\right)\right]$ (a function of *p^B^* and *d^S^*) can be approximated using Taylor series expansions, as described in the Methods section (method 4). Method 4 can also be used when $\text{φ}=\left\{\alpha ,p^{B}\right\}$.

#### Example 5: Net Benefit Includes Joint Nonlinear Functions of Correlated Focal and Nonfocal Parameters

Suppose we wish to evaluate the value of collecting routine data on mortality under current practice (i.e., without fluid resuscitation). Here, φ={α}, so that ${\text{φ}}^{C}=\left\{{\text{p}}^{\text{B}},p^{L},{\text{q}}^{\text{S}},{\text{q}}^{M},{\text{d}}^{\text{M}},d^{S}\right\}$. In this case, by defining

$$\beta_{1}(\theta )=\frac{\exp(\alpha )}{\left(1+\exp(\alpha )\right)}(Wq^{M}-C^{H}-p^{L}\left(\frac{p^{B}\exp(d^{S})}{\left(1+p^{B}\exp(d^{S})\right)}\right)(Wq^{S}+C^{S}))$$

$$\beta_{2}(\varphi^{C})=p^{L}\left(\frac{p^{B}\exp(d^{S})}{\left(1+p^{B}\exp(d^{S})\right)}\right)(Wq^{S}+C^{S})$$

$${h_{1,}}_{j}(\theta )=\left(\frac{\exp(\alpha +d_{j}^{M})}{\left(1+\exp(\alpha +d_{j}^{M})\right)}\right)(Wq^{M}-C^{H})$$

$$\begin{array}{c} h_{2,j}(\theta )=\left(\frac{\exp(\alpha +d_{j}^{M})}{\left(1+\exp(\alpha +d_{j}^{M})\right)}\right)\left(\frac{p^{B}\exp(d^{S})}{\left(1+p^{B}\exp(d^{S})\right)}\right), \end{array}$$

the net benefit can be expressed as

$$B_{j}({\text{φ}}^{C},\text{φ})=\beta_{1}\left(\theta \right)-{h_{1,}}_{j}(\theta )+{h_{2,}}_{j}(\theta )\beta_{2}\left({\text{φ}}^{C}\right)-C_{j}^{F}.$$

Because *d^M^* and *d^S^* are correlated, none of the methods previously described can be used to estimate $\underset{{\text{φ}}^{C}}{E}\left[{h_{2,}}_{j}(\theta )\right]$. However, restricted cubic splines $\xi_{j}\left(\alpha \right)$ can be constructed (method 5) such that

$$\xi_{j}\left(\alpha \right)\approx \underset{{\text{φ}}^{C}|\alpha }{E}\left[h_{2,j}\left({\text{φ}}^{C},\alpha \right)\right].$$

Then, EVPPI[^α^] can be approximated by

$$\begin{array}{c} \mathrm{EVPPI}\left[\alpha \right]=\underset{\alpha }{E}\left[\underset{j}{\max}\left\{\underset{{\text{φ}}^{C}|\alpha }{E}\left[B_{j}({\text{φ}}^{C},\text{φ})-B_{j*}({\text{φ}}^{C},\text{φ})\right]\right\}\right]\\ \approx \underset{\alpha }{E}\left[\underset{j}{\max}\left\{E\left[\beta_{2}\left({\text{φ}}^{C}\right)\right]\left(\xi_{j}\left(\alpha \right)-\xi_{j*}\left(\alpha \right)\right)+C_{j}^{F}-C_{j*}^{F}\right\}\right] \end{array}.$$

The method described for this example can also be used when φ is any single element of $\left\{d^{S},d^{M},p^{B}\right\}$.

## Results

### Evidence Synthesis and Cost-Effectiveness Analysis

We carried out a Bayesian evidence synthesis, using WinBUGS version 1.4.3,^19^ to estimate posterior distributions for the treatment effect parameters *d^M^* and *d^S^* based on the data from the 3 pilot studies (details in the supplementary appendix). A Markov chain Monte Carlo (MCMC) simulation with a burn-in of 50 000 (the number of samples required for convergence of multiple chains) followed by simulation of 5 million parameter value sets (chosen based on the memory limits of the computer) was used to represent joint parameter uncertainty, which forms the prior for the EVPPI analysis.

Table 4 gives posterior parameter means and correlations, based on the pilot studies listed in supplementary appendix A2. The impact of saving a life on the subsequent risk of NS, *d^S^*, is correlated with the effect of each treatment on mortality, *d^M^*. The treatment effects on mortality are also correlated with each other. This is because *d^S^* and *d^M^* are jointly estimated from the pilot studies, where the observed proportion of NS depends on both *d^S^* and *d^M^* (supplementary appendix A2). No other correlations exist between model parameters because they are estimated from independent data sources.

**Table 4:** Results of MCMC Simulation, with Unconditional Means and Correlations between Parameters

		Posterior Mean and Variance		Correlations								
Parameter	Description	Mean	Variance	α	${\text{d}}_{2}^{\text{M}}$	${\text{d}}_{3}^{\text{M}}$	${\text{d}}_{4}^{\text{M}}$	*P^B^*	*d^S^*	*p^L^*	*q^M^*	*q^S^*
α	Baseline mortality (logit scale)	−1.07	0.11	1	0	0	0	0	0	0	0	0
$d_{2}^{M}$	Effect of albumin on mortality	−0.31	0.51	0	1	0.64	0.44	0	0.25	0	0	0
$d_{3}^{M}$	Effect of saline on mortality	−2.34	0.64	0	0.64	1	0.51	0	0.27	0	0	0
$d_{4}^{M}$	Effect of gelofusine on mortality	−0.19	1.81	0	0.44	0.51	1	0	0.27	0	0	0
*P^B^*	Baseline NS	0.10	0.04	0	0	0	0	1	0	0	0	0
*d^S^*	Change to NS risk in “saved” patients	1.88	4.52	0	0.25	0.27	0.27	0	1	0	0	0
*p^L^*	Probability that short-term NS proves permanent	0.50	0.08	0	0	0	0	0	0	1	0	0
*q^M^*	QALYs gained by those who survive and are NS-free	19.99	24.97	0	0	0	0	0	0	0	1	0
*q^S^*	QALY loss from NS	5.38	7.94	0	0	0	0	0	0	0	0	1

Note: MCMC = Markov chain Monte Carlo; NS = neurological sequelae; QALY = quality-adjusted life-year.

Albumin is the only treatment for which the 95% credible interval for the log-odds ratio relative to no fluids does not include 0 (no effect). Appropriate cost-effectiveness thresholds in an African context are less well established than in regions such as the United Kingdom. We therefore explored a range for the willingness-to-pay threshold (WTP) of 0 to 4000 USD/quality-adjusted life-year (QALY) when calculating net benefit. Based on the pilot data and the synthesis model, the most cost-effective option is albumin if WTP is >$269/QALY and gelofusine otherwise (there are no choices of threshold for which saline or no fluid is the optimal treatment). Around the “switching” threshold of $269/QALY, there is considerable uncertainty about the most cost-effective treatment. For example, although gelofusine has the highest expected net benefit at a threshold of $250/QALY, there is only an 18% probability that it is the most cost-effective at that threshold, compared with 54% for albumin, 7% for saline, and 21% for no fluids. Results are more certain at higher thresholds; at $1000/QALY, for example, albumin has the highest net benefit with a 92% probability.

### Value of Information

All calculations were performed using the software package R (version 2.15). For the effectiveness parameters *d^M^* and *d^S^*, these samples were generated using MCMC simulation from the evidence synthesis model. For the remaining parameters, samples were generated in R directly. Computations were performed on a PC with an Intel i5 processor and 3 Gb of RAM.

**Figure 2:**
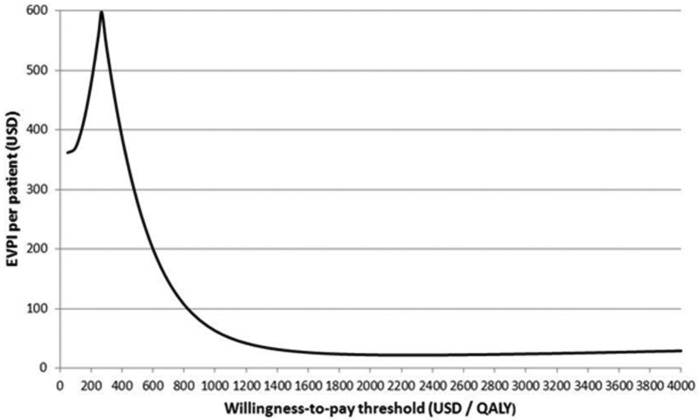
Expected value of perfect information for the fluid resuscitation case study, as a function of the decision maker’s willingness-to-pay threshold over the range $50 to $4000 per quality-adjusted life-year.

The EVPI is highest around the threshold at which the optimal treatment changes, where the decision is most uncertain and sensitive to additional information (Figure 2). Another influence on the EVPI/WTP relationship is that, at low threshold values, NS costs are high enough for additional information on NS-related parameters (*q^S^, d^S^, p^L^, p^B^*) to potentially change the decision. This effect can be verified by calculating the EVPPI for different model parameters. Table 5 illustrates EVPPI estimates for different subsets of parameters, each calculated in a single simulation step using the methods described above, assuming a WTP of $250/QALY. Methods that involve replacing nonfocal parameters or functions of them, with their unconditional means in the net benefit function (methods 1–3), can be used to estimate the benefit of knowing individual parameters with certainty in 3 cases: *p^L^, q^M^*, and *q^S^*. This benefit is large for the first 2 parameters but 0 for *q^S^* (the QALY loss associated with persistent NS).

**Table 5:** Estimates of EVPPI for a Range of Parameter Subsets, Based on a Willingness-to-Pay Threshold of $250/QALY

			Computation Times	
Focal Parameter	Method for Single-Step Estimation	EVPPI ($)	Nested 106 × 103	One Step
All	NA	561 (EVPI)	22 s	
dM, α, pB, dS, pL	Method 1	546	26 min	9 s
dM, α, pB, dS, qM	Method 2	415	26 min	10 s
qM	Method 3	73	32 min	21 s
qS	Method 3	0	32 min	25 s
pL	Method 3	239	31 min	20 s
dM, dS	Method 4	342	31 min	1 min 24 s
pB	Method 5	87	31 min	46 s
α	Method 5	0	31 min	44 s
dS	Method 5	243	31 min	46 s
$d_{2}^{M}$(saline)	Method 5	38	32 min	47 s
$d_{3}^{M}$(albumin)	Method 5	$14	32 min	59 s
$d_{4}^{M}$(gelofusine)	Method 5	$24	32 min	46 s

Note: Computation was carried out on a desktop PC with 8 Gb RAM and an Intel i5-2400 processor. EVPI = expected value of perfect information; EVPPI = expected value of perfect parameter information; QALY = quality-adjusted life-year.

**Figure 3:**
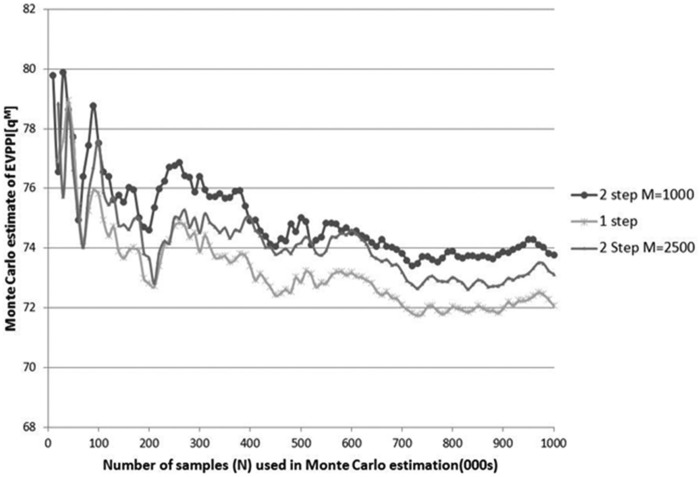
Monte Carlo estimates of the expected value of perfect parameter information of *q^M^* (quality-adjusted life-years gained through neurological sequelae–free survival) derived using nested versus 1-step Monte Carlo simulation. Estimates were calculated from *N* values sampled from the joint posterior distribution of all parameters. One-step estimation was carried out using method 3. Nested simulation was carried out by subsampling (from the *N* samples) *M* values of the nonfocal parameters *N* times (once for each sampled value of the focal parameter) to estimate conditional expected net benefit.

The accuracy of EVPPI estimated using methods 1 to 3 depends on the precision of the estimates of the unconditional parameter means, which in turn will depend on the size of the sample generated from the posterior distribution. The point at which sample size is sufficient can be assessed by plotting the relationship between the estimate of EVPPI and the sample size used to derive it. Figure 3 illustrates the number of outer simulations needed for EVPPI estimates to converge in the case where $\text{φ}=q^{M}$ and compares nested and single-step simulation. Sample sets of increasing size were used to estimate $\mathrm{EVPPI}\left({\text{q}}^{\text{M}}\right)$ using the 1-step approach of method 3 and the 2-step approach with 1000 or 2500 inner simulations for each outer simulation. The (upward) bias resulting from an inadequate number of inner simulations can be clearly seen: with 1000 inner samples per outer simulation, the bias is about 3% of the total EVPPI. Nested simulation is also far slower than 1-step simulation; Table 5 gives computation times using each approach, showing the former to be at least 25 times slower when it is based on 1000 simulations in the inner loop, and no allowance is made for correlations between parameters (any attempt to incorporate correlations into nested simulation would further increase the speed advantage of the 1-step approach).

**Figure 4:**
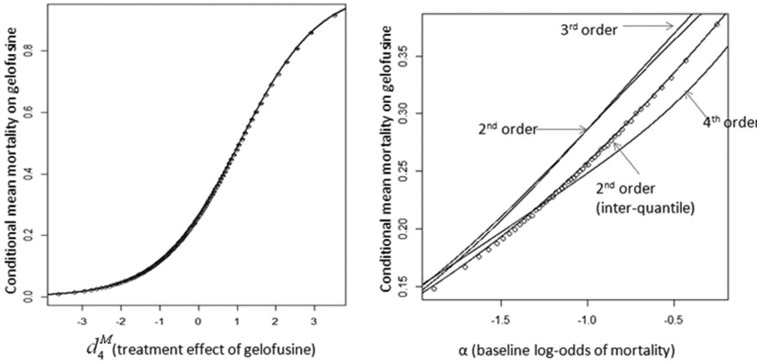
Taylor series approximations to expected mortality on gelofusine conditional on 1) treatment effect of gelofusine (Figure 4.1) and 2) baseline log-odds of mortality (Figure 4.2), for estimation of expected value of perfect parameter information using method 4. Circles represent direct estimates of conditional means, and lines illustrate alternative Taylor series approximations (indistinguishable in the first case).

**Figure 5:**
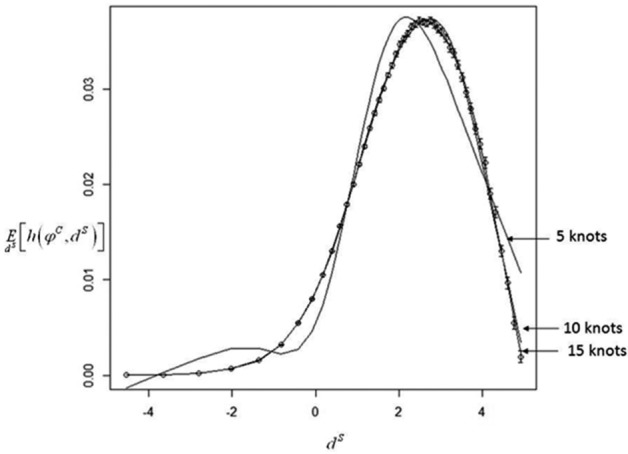
Restricted cubic splines approximating the expectation of *h*(ϕ^*C*^, *d^S^*) conditional on *d^S^* (impact of successful treatment on risk of neurological sequelae) to allow 1-step estimation of $\mathrm{EVPPI}\left({\text{d}}^{\text{S}}\right)$(example 5). Circles represent estimates of the conditional expectation across the plausible range of *d^S^*. Error bars represent 95% confidence intervals for each sample mean. The graph shows best-fit splines using 5, 10, and 15 knots.

Methods 4 and 5 involve constructing functions that give approximate values for conditional means. The accuracy of EVPPI estimates will therefore depend on the goodness of fit of the approximating function, which can be explored graphically. Figure 4 illustrates Taylor series approximations for $E\left[\frac{\exp(\alpha +d_{4}^{M})}{\left(1+\exp(\alpha +d_{4}^{M})\right)}\right]$, the expected mortality with gelofusine conditional on perfect information for 1) a known treatment effect $d_{4}^{M}$ and 2) a known baseline mortality α. In the first case, the second-order approximation is extremely accurate and cannot be discernibly improved by higher-order terms or the use of interquartile means. In the second case, the second-order approximation is noticeably inaccurate, the fourth-order approximation is still inaccurate but less so, and the approximation based on interquartile means is extremely accurate. However, using the more accurate approximation changes estimated EVPPI by only 1%, from $345 to $342 per person (the latter figure, based on the more accurate approximation, is the one quoted in Table 5). Figure 5 illustrates, in the case where $\text{φ}=d^{S}$ (example 5), how increasing the number of knots in a spline improves its accuracy, although the difference between 10 and 15 knots is negligible. We found that estimates of EVPPI were extremely stable as the number of knots increased; results in Table 5 were calculated using 10 knots, although results with 6 knots differed by less than 1% in all cases.

## Discussion

We have presented a range of methods to avoid the need for nested simulation in EVPPI calculations. The advantages of a 1-step simulation strategy for the computation of EVPPI include unbiased estimation and computational efficiency. It is well known that the inner simulation step can be avoided when the net benefit function is linear or multilinear in the focal parameters (methods 1 and 2).^4,9^ We have extended these methods to a broader range of net benefit functions by identifying functions of the focal parameters in which the net benefit function is (multi)linear (method 3), which has been used previously,^17^ but the conditions in which the method can be used have not been set out before, and it is not commonly employed. This may be because it requires the net benefit function implied by the economic model to be stated explicitly and to be manipulated algebraically, whereas EVPPI analyses commonly treat the model as a “black box” generating net benefit estimates. Ades and others^9^ proposed the use of Taylor series approximations to the inverse-logit to approximate conditional expectations. We have set out the situations in which a Taylor series approximation can be used in general (method 4), adapted the approximation for the inverse-logit to improve accuracy, and given the formulae for other commonly used functions. We are unaware of any previous work that has proposed using spline techniques (method 5).

Our use of Taylor series expansions (method 4) and splines (method 5) can be seen as conceptually similar to existing statistical meta-modeling approaches that have been advocated in place of nested simulation, such as Gaussian process emulation.^20^ Tappenden and others^21^ reviewed potential meta-modeling methods and noted that although they may permit flexible estimation of EVPPI with reasonable accuracy, these methods require substantial specialist expertise. The key difference between meta-modeling and our approximation techniques is that we model components of the net benefit function, rather than the entire economic model. As a result, the methods we present require less technical expertise to implement and review. Furthermore, these methods may be useful for models with a large number of parameters, when Gaussian process emulation can be unfeasible.^7^

The accuracy of approximations to the conditional expectation of the net benefit function should always be checked by comparing approximate with actual values over a plausible range of the focal parameters, as we illustrate in Figure 4. We found that Taylor series expansion around the unconditional mean of the nonfocal parameter could be markedly inaccurate if that parameter had a high variance, even if higher-order terms were included in the expansion. For such situations, we outline an approach averaging over Taylor series expansions around each of the interquartile means, which greatly improves the accuracy of this approach. We found spline regressions were able to provide close approximations to the conditional mean, particularly if enough knots were chosen. The accuracy of spline approximations is largely driven by the accuracy of the bin means to which they are fitted and the coverage of bin means across the plausible range of the focal parameter. In particular, simulated values will be sparse for values of the focal parameter that are less likely. If the optimal treatment changes for these values, it is particularly important to consider the accuracy of the spline approximation of the expectation conditional on these values. The density of conditional mean estimates across the range of the focal parameter can be increased by using more bins, although this will lead to larger sampling errors for each bin mean if the total number of samples is kept constant. The number of samples *N*_θ_ used for spline estimation should be large enough to support enough bins across the plausible range of the focal parameter while ensuring sufficiently low standard errors for each bin mean.

A particular challenge for EVPPI calculations occurs when correlations exist between components of ${\text{φ}}^{C}$ and components of φ.^5^ In this situation, values at each inner simulation need to be sampled from the conditional distribution of ${\text{φ}}^{C}$ ; otherwise, EVPPI estimates will be downwardly biased. One approach would be to carry out a separate MCMC simulation for each realization from the outer simulation. This will give samples of ${\text{φ}}^{C}$ from the correct distribution but at considerable computational expense. A more efficient approach may be to assume a parametric joint distribution for the correlated parameters, from which a conditional sampling distribution can be derived at each inner simulation. However, this may introduce an additional source of bias. One-step estimation of EVPPI using spline approximations allows us to side step this problem.

The purpose of VoI is to guide decisions on research prioritization, so that it is necessarily conducted in situations in which current information is inadequate and assumptions may be proven quite wrong by later research. The goal is to reflect as fairly as possible current information, however sparse that might be. The credibility of VoI estimates therefore depends on the credibility of the cost-effectiveness model on which they are based and how accurately prior distributions reflect prestudy parameter uncertainty, which requires guidance from clinicians and decision makers regarding assumptions made in the model and plausible alternatives. Decision makers might require the model to be extended to allow for different severities of NS or the health utility loss due to short-term NS to be included. We have assumed fixed treatment effects across studies and used pilot study data to estimate them. These data were the only evidence available to us and were insufficient to estimate less restrictive models; thus, it is possible that we have underestimated uncertainty in treatment effect estimates. Furthermore, use of pilot study data may introduce bias, as their design and conduct may not be as rigorous as a full RCT. Very little information was available on the long-term costs and health consequences of NS, and the values chosen for this analysis were therefore not evidence based but chosen to ensure nonzero EVPPI estimates for these parameters. When existing evidence is sparse and/or of poor quality, as with long-term NS in our case study, it is important to be able to somehow characterize the extent of the uncertainty, as VOI will be highly sensitive to this. One possibility is to incorporate formal expert elicitation of plausible parameter values, together with opinion on the possible degree of bias in the existing evidence base.^22^ Finally, we would advocate the use of sensitivity analyses to explore the sensitivity of VoI results to modeling assumptions,^17^ as would be conducted as standard practice for cost-effectiveness analysis.^23^

The focus of this article is to illustrate methods for 1-step estimation of EVPPI. Because of the limitations set out above, we do not intend to draw conclusions about research priorities in the area of fluid resuscitation. Nevertheless, the case study illustrates how EVPPI calculations can be informative when considering the relative merits of efficacy trials compared with longer-term epidemiological studies or when selecting outcome measures and treatments to include in a proposed trial. For example, there was considerable debate following the pilot studies around the inclusion of albumin in the FEAST trial, driven by perceptions that this treatment was too costly for use in Africa. Our EVPPI analysis suggests that there was considerable value to including this arm, although the caveats mentioned above apply. The results from the FEAST trial have recently been published,^16^ with robust but unexpected findings (harm was demonstrated in the fluid resuscitation arms).

A key limitation of our approach is that the methods rely on being able to explicitly state the net benefit function implied by the model. We would argue that there are clear benefits in explicitly setting out the net benefit function, in terms of transparency, understanding, and communication of assumptions, as well as the reduced computational burden for VoI calculations, and would always recommend doing this unless the decision model is unavoidably too complex to allow it. We have presented the use of univariate splines in our case study, which can be used only if there are no nonlinear functions of multiple correlated focal parameters in the net benefit function. When this is not the case (for example, if we were to consider 2- or 3-arm trials in the fluid replacement example), then the spline approach (method 5) could be implemented through the use of multivariate adaptive regression splines.^7^

In this article, we have focused on EVPPI; however, the methods presented here can also be used in the calculation of the expected value of sample information (EVSI). EVSI is more computationally intensive than EVPPI because it requires an extra layer of simulation required to simulate new data from a given new study design. Furthermore, if we wish to optimize over potential new study design factors, then EVSI will need to be evaluated repeatedly, which will substantially increase the computational burden. Ades and others^9^ set out algorithms for the estimation of EVSI in a number of situations, all of which require nested Monte Carlo simulation unless the net benefit function satisfies the conditions required for use of methods 1 and 2. The methods presented here extend the circumstances in which we can avoid the inner simulation step for EVSI, hence improving computing times and thereby making the use of EVSI feasible in a wider range of situations.

## Supplementary Material

Supplementary material

## Supplementary Material

Supplementary material
