# Supplementary material for: Strategies for Efficient Computation of the Expected Value of Partial Perfect Information
Source: Med Decis Making. 2014 Jan 21;34(3):327–42. doi: 10.1177/0272989X13514774 (PMC4948652; doi:10.1177/0272989X13514774)
Supplement: Supplementary material [file DS_10.11770272989X13514774_Appendix.pdf]

Appendix (for online publication only)

A1: Taylor series expansions for functions commonly found in net benefit functions

Our aim is to find an approximation for the expected value of a function h(X) of a random variable X.

The nth order Taylor series expansion of h(X) around a given constant $\tilde{X}$, $h_{Tn}\left(X,\tilde{X}\right)$, is:

$$h_{Tn}\left(X,\tilde{X}\right)=\sum_{i=0}^{n}\frac{\left(X-\tilde{X}\right)^{i}}{i!}\frac{d^{i}h\left(\tilde{X}\right)}{dX^{i}}$$

We divide the distribution of X into J quantiles, by defining a vector $q=(q_1,...q_{J+1})$such that:

$$P(q_j \le X \le q_{j+1}) = 1/J \quad \forall \text{ j}$$

We define $E_j^Q[X]$ as the mean of all the values of X that lie in the jth quantile, so that:

$$E_j^Q\left[X\right]=E[X \mid q_j \le X \le q_{j+1}] \, , 1 \le j \le J$$

We define $H_j^Q[X]$ as the expected value of h(X) conditional on X lying within the jth quantile. Using the associated expectation $E_j^Q[X]$ as the point around which to expand a Taylor series approximation, we have:

$$
\begin{aligned}
H_j^Q[X] &\simeq E\left[h_{Tn}\left(X, E_j^Q[X]\right) \mid q_j \le X \le q_{j+1}\right] \\
&\simeq \sum_{i=0}^{n} E\left[\frac{\left(X - E_j^Q[X]\right)^i}{i!} \mid q_j \le X \le q_{j+1}\right] \frac{d^i h\left(E_j^Q[X]\right)}{dX^i} \\
&\simeq \sum_{i=0}^{n} M_{i,j}^Q[X] \frac{d^i h\left(M_{1,j}^Q[X]\right)}{dX^i}
\end{aligned}
$$

where $M_{i,j}^Q[X] = E\left[\frac{\left(X - E_j^Q[X]\right)^i}{i!} \mid q_j \le X \le q_{j+1}\right]$

Note that $M_{1,j}^Q[X] = E_j^Q[X]$. The unconditional mean of h(X), H(X), can then be defined in terms of the conditional means $H_j^Q[X]$:

$$
\begin{aligned}
H[X] &= \sum_{j=1}^{J} P\left(q_j \le X \le q_{j+1}\right) H_j^Q[X] \\
&= \frac{1}{J} \sum_{j=1}^{J} H_j^Q[X]
\end{aligned}
$$

This gives an approximation for the required mean of h(X) as a function of the $M_{i,j}^Q[X]$. In particular, when J=1 we have $M_{1,1}^Q[X] = E[X]$ and $M_{2,1}^Q[X] = Var[X]$, and the approximation reduces to: $H(X) \simeq h(E[X]) + \frac{1}{2}\frac{d^2 h\left(E[X]\right)}{dX^2} Var[X] + \ldots + \frac{1}{n!}\frac{d^n h\left(E[X]\right)}{dX^n} E\left[\left(X - E[X]\right)^n\right]$

A Taylor series approximation for the inverse logit function

The inverse logit function is defined by

$$
h(X) = \frac{e^X}{1 + e^X}
$$

Defining $y = e^X$, and using the chain and product rules for differentiation, we have:

$$\frac{dh}{dx} = \frac{dh}{dy}.\frac{dy}{dx} = \left(\frac{1}{1+y} - \frac{y}{(1+y)^2} \right) y$$
$$= h - h^2$$

$$\frac{d^2h}{dx^2} = \frac{dh}{dx}\frac{d}{dh}\left(h-h^2\right) = (h-h^2)(1-2h)$$
$$= 2h^3 - 3h^2 + h$$

$$\frac{d^3h}{dx^3} = \frac{dh}{dx}\frac{d}{dh}\left(2h^3-3h^2+h\right) = \left(h-h^2\right)\left(6h^2-6h+1\right)$$
$$= -6h^4 + 12h^3 - 7h^2 + h$$

$$\frac{d^4h}{dx^4} = \frac{dh}{dx}\frac{d}{dh}\left(-6h^4+12h^3-7h^2+h\right) = \left(h-h^2\right)\left(-24h^3+36h^2-14h+1\right)$$
$$= 24h^5 - 60h^4 + 50h^3 - 15h^2 + h$$

From which we can derive the 2nd , 3rd and 4th order approximations for E[h(X)]. Defining

$H = h(E[X])$:

$$E\left[h_{T2}(X)\right] = H + \frac{2H^3 - 3H^2 + H}{2} Var[X]$$

$$E\left[h_{T3}(X)\right] = E\left[h_{T2}(X)\right] + \frac{-6H^4 + 12H^3 - 7H^2 + H}{6} E\left[\left(X - E[X]\right)^3\right]$$

$$E\left[h_{T4}(X)\right] = E\left[h_{T3}(X)\right] + \frac{24H^5 - 60H^4 + 50H^3 - 15H^2 + H}{24} E\left[\left(X - E[X]\right)^4\right]$$

B Taylor series expansion for the inverse complementary log-log function

The inverse complementary log-log function is defined by:

$$h(X) = 1 - e^{-e^X}$$

Defining $y = e^X$, and using the chain and product rules for differentiation, we have:

$$\frac{dh}{dx} = \frac{dh}{dy}.\frac{dy}{dx} = y\frac{d}{dy}\left(1-e^{-y}\right)$$
$$= ye^{-y}$$

$$\frac{d^2h}{dx^2} = \frac{dy}{dx}\frac{d}{dy}\left(ye^{-y}\right) = y\left(e^{-y} - ye^{-y}\right)$$
$$= e^{-y}\left(y - y^2\right)$$

$$\frac{d^3h}{dx^3} = \frac{dy}{dx}\frac{d}{dy}\left(\left(y - y^2\right)e^{-y}\right) = y\left((1-2y)e^{-y} - \left(y - y^2\right)e^{-y}\right)$$
$$= e^{-y}\left(y - 3y^2 + y^3\right)$$

$$\frac{d^4h}{dx^4} = \frac{dy}{dx}\frac{d}{dy}\left(e^{-y}\left(y - 3y^2 + y^3\right)\right) = y\left(\left(1 - 6y + 3y^2\right)e^{-y} - e^{-y}\left(y - 3y^2 + y^3\right)\right)$$
$$= e^{-y}\left(\left(y - 6y^2 + 3y^3\right) - \left(y^2 - 3y^3 + y^4\right)\right)$$
$$= e^{-y}\left(y - 7y^2 + 6y^3 - y^4\right)$$

From which we can derive the 2nd , 3rd and 4th order approximations for E[h(X)]. Defining

$Y = y(E\left[X\right])$:

$$E\left[h_{T2}\left(X\right)\right] = 1 - e^{-Y} + \frac{e^{-Y}\left(Y - Y^2\right)}{2}Var[X]$$

$$E\left[h_{T3}\left(X\right)\right] = E\left[h_{T2}\left(X\right)\right] + \frac{e^{-y}\left(y - 3y^2 + y^3\right)}{6}E\left[\left(X - E[X]\right)^3\right]$$

$$E\left[h_{T4}\left(X\right)\right] = E\left[h_{T3}\left(X\right)\right] - \frac{e^{-y}\left(y - 7y^2 + 6y^3 - y^4\right)}{24}E\left[\left(X - E[X]\right)^4\right]$$

A2 Bayesian network meta-analysis of pilot study data.

Evidence Synthesis Model

Table A1 gives the results from the 3 pilot studies that represent the available evidence on fluid resuscitation for cerebral malaria. We developed a model to synthesise this evidence base in order to inform an economic evaluation and value of information analysis. We assume that the survival and NS status of participants in each of the study arms in table A1 are independently identically distributed Bernouilli variables, so that the data can be modelled as samples from Binomial distributions. Let r_{ik}^{M} and r_{ik}^{NS} be the numbers in arm k of study i who die or experience NS respectively. Then,

$$r_{ik}^{M} \sim binomial\left(p_{ik}^{M}, N_{ik}\right)$$

$$r_{ik}^{A} \sim binomial\left(p_{ik}^{A}, N_{ik} - r_{ik}^{M}\right)$$

where p_{ik}^{M} is the probability of death in arm k of trial i, and p_{ik}^{A} is the probability that survivors in that arm have adverse NS at 28 days (the follow-up period of the pilot studies). We assume a logistic model for the effect of treatment on p_{ik}^{M} :

$$\text{logit}(p_{ik}^{M}) = \alpha_i + \delta_{i,k}^{M}$$

Here α_i is the log-odds of the rate of mortality in the baseline arm for trial i , and $\delta_{i,k}^{M}$ is the impact on mortality of the treatment for arm k of study i, relative to the baseline (implying that $\delta_{i,1}^{M} = 0$) . We fit a Mixed Treatment Comparison (or Network Meta-Analaysis) model (15) assuming a fixed

relative treatment effect (due to lack of data with which to fit a random effects model). The Mixed Treatment Comparison model assumes consistency amongst relative treatment effects, so that:

$$\delta_{i,k}^{M} = d_{j(i,k)}^{M} - d_{j(i,1)}^{M}$$

where $j(i,k)$ is an index for the treatment received in arm k of trial i (1= no fluid, 2 =saline, 3=albumin, 4=gelofusine) and $d_{j(i,k)}^{M}$ is the impact of that treatment, relative to treatment 1. We set $d_{1}^{M} = 0$.

There is an additional outcome of interest in our analysis, NS. Both outcomes are influenced by the severity of the initial reaction to the disease. Therefore, there is a link between the likelihood of each outcome, which could be represented in several ways. We assume that those who are 'saved' from death by treatment have a separate risk of NS, which may be either less than, or more than, the risk of NS in those who would survive under any treatment. This implies that:

$$p_{i,k}^{A} = \frac{p_{i,1}^{A}(1 - p_{i,1}^{M}) + p^{S}(p_{i,1}^{M} - p_{i,k}^{M})}{(1 - p_{i,k}^{M})}$$

where p^{S} is the probability of NS in those who are 'saved' by the more effective treatment. We define d^{S} as the difference, on the log-odds scale, in the probability of NS in those who would survive without fluids and those who are saved by fluid resuscitation, which implies that:

$$p^S = \left(\frac{p_1^A \exp(d^S)}{(1 + p_1^A \exp(d^S))} \right)$$

A consequence of our model structure is that, given results from trials including both mortality and NS, we would expect the posterior distributions of d^S and d^M to be correlated.
