# Supplementary material for: Strategies for Efficient Computation of the Expected Value of Partial Perfect Information
Source: Med Decis Making. 2014 Jan 21;34(3):327–42. doi: 10.1177/0272989X13514774 (PMC4948652; doi:10.1177/0272989X13514774)
Supplement: Supplementary material [file DS_10.11770272989X13514774_Table_A1.pdf]

Trial	Fluid given	Number of participants	deaths	Neurological Sequelae
1: Akech 2006	Albumin	40	1	3
	Gelofusine	40	4	1
2: Maitland 2005(severe acidosis)	Albumin	23	2	3
	Saline	26	8	1
3: Maitland 2005 (moderate acidosis)	Albumin	33	0	3
	Saline	35	3	1
	No fluids*	33	2	0
4: Maitland 2003	Albumin	15	0	0 / NA
	Saline	26	4	0 / NA

Table A1: results from the pilot studies.

*5 children randomised to no fluids were switched to Saline during the trial.
